# Evolution of the Vertebrate Paralemmin Gene Family: Ancient Origin of Gene Duplicates Suggests Distinct Functions

**DOI:** 10.1371/journal.pone.0041850

**Published:** 2012-07-25

**Authors:** Greta Hultqvist, Daniel Ocampo Daza, Dan Larhammar, Manfred W. Kilimann

**Affiliations:** 1 Department of Neuroscience, Unit of Molecular Cell Biology, Uppsala University, Uppsala, Sweden; 2 Department of Neuroscience, Unit of Pharmacology, Uppsala University, Uppsala, Sweden; University of Lausanne, Switzerland

## Abstract

Paralemmin-1 is a protein implicated in plasma membrane dynamics, the development of filopodia, neurites and dendritic spines, as well as the invasiveness and metastatic potential of cancer cells. However, little is known about its mode of action, or about the biological functions of the other paralemmin isoforms: paralemmin-2, paralemmin-3 and palmdelphin. We describe here evolutionary analyses of the paralemmin gene family in a broad range of vertebrate species. Our results suggest that the four paralemmin isoform genes (*PALM1*, *PALM2, PALM3* and *PALMD*) arose by quadruplication of an ancestral gene in the two early vertebrate genome duplications. Paralemmin-1 and palmdelphin were further duplicated in the teleost fish specific genome duplication. We identified a unique sequence motif common to all paralemmins, consisting of 11 highly conserved residues of which four are invariant. A single full-length paralemmin homolog with this motif was identified in the genome of the sea lamprey *Petromyzon marinus* and an isolated putative paralemmin motif could be detected in the genome of the lancelet *Branchiostoma floridae*. This allows us to conclude that the paralemmin gene family arose early and has been maintained throughout vertebrate evolution, suggesting functional diversification and specific biological roles of the paralemmin isoforms. The paralemmin genes have also maintained specific features of gene organisation and sequence. This includes the occurrence of closely linked downstream genes, initially identified as a readthrough fusion protein with mammalian paralemmin-2 (Palm2-AKAP2). We have found evidence for such an arrangement for paralemmin-1 and -2 in several vertebrate genomes, as well as for palmdelphin and paralemmin-3 in teleost fish genomes, and suggest the name paralemmin downstream genes (PDG) for this new gene family. Thus, our findings point to ancient roles for paralemmins and distinct biological functions of the gene duplicates.

## Introduction

Paralemmins are a protein family with four previously characterized isoforms in humans and mice: paralemmin-1, paralemmin-2, paralemmin-3 and palmdelphin. The first three are anchored to the plasma membrane through prenylation and di-palmitoylation of a C-terminal cysteine cluster (CaaX motif), whereas palmdelphin is predominantly expressed as a splice variant that lacks the CaaX motif, thereby becoming a cytosolic protein [1]. Paralemmin-1 stimulates cell expansion and the extension of filopodia and processes in fibroblasts, and the formation of filopodia, neurites and dendritic spines in neurons [2], [3], [4]. Increased expression of paralemmin-1 has been correlated with tumor progression, invasion or metastatic potential [5], [6], [7]. Paralemmins associate with lipid rafts and have been proposed to function as adaptors between membrane proteins or with the cortical cytoskeleton [4]. This notion is supported by observations of binding between paralemmin-1 and the dopamine receptor D3 [8] and between paralemmin-3 and SIGIRR (single immunoglobulin IL-1 receptor-related molecule) [9]. Human and mouse paralemmin-2 can also be expressed as a fusion protein with the product of a downstream gene, the A kinase anchor protein 2 gene (*AKAP2*) through transcriptional readthrough and differential splicing [10].

The presence of four paralemmin genes (*PALM1, PALM2, PALM3* and *PALMD*) on four distinct chromosomes in mammals suggested that this gene family might have arisen from a single ancestral gene through the two rounds of tetraploidization early in vertebrate evolution referred to as 2R [11], [12], [13]. The lineage leading to the lancelets diverged from the rest of the chordate lineage before the 2R events, but shares several attributes common to all chordates like a hollow dorsal neural tube and a notochord [14]. These facts, together with the sequenced genome of the Florida lancelet *Branchiostoma floridae* [11], [15], make this lineage highly interesting in the evolution of complex nervous systems. It has been suggested that 2R provided many new genes that have contributed to vertebrate features such as a complex nervous system, the neural crest, jaws, limbs etc. [16]. A third tetraploidization event, called 3R, occurred in the teleost fish lineage approximately 350 million years ago [17], [18], [19], which might have generated additional paralemmin diversity. Many gene families, essential for the function and development of the brain and nervous system, have been found to have expanded in this way [20], [21], [22], [23], [24], [25], [26].

There are several reasons why the paralemmin gene family merits closer evolutionary study of sequence features. The known paralemmin proteins are characterized by their predicted intrinsic disorder and extensive sequence regions with low inter-species conservation. Conserved sequence features common to all paralemmins have not been characterized until the present study. These may be utilized to explore interactions of paralemmins with other proteins involved in the many functions of the paralemmins. Further, the C-termini of paralemmin-2 and palmdelphin show differential splicing and the *PALM2* and *AKAP2* genes produce a peculiar fusion product, why it is interesting to investigate if similar features are found in other paralemmin gene family members.

Here we have investigated the evolution of the paralemmin family by analyzing the genomes of a wide selection of vertebrate species, as well as the lancelet *Branchiostoma floridae*. To determine the evolutionary relationships between the family members, we have also analyzed gene families that show conserved synteny with the paralemmin genes. In addition to the sometimes co-transcribed *AKAP2*, we included the neighboring families of sphingosine-1–phosphate receptors (*S1PR*), plasticity related genes (*PRG*) (also called lipid phosphate phosphatase related proteins), ATP-binding cassette subfamily A (*ABCA*), calponin (*CNN*), and polypyrimidine tract-binding protein (*PTBP*) in the analysis.

The combined information from sequence-based phylogenies as well as chromosomal locations of the paralemmin genes and the adjacent gene families allow us to conclude that the paralemmin family expanded in the early vertebrate genome duplications and thus that all four paralemmin isoforms have been retained for more than 450 million years of vertebrate evolution, suggesting a unique functional role for each family member.

## Methods

### Database searches and identification of paralemmin genes

The human paralemmin family sequences were used as queries in tblastn searches [27] of the Ensembl database [28] (www.ensembl.org) in the following vertebrate genomes: human (*Homo sapiens*), mouse (*Mus musculus*), chicken (*Gallus gallus*), Western clawed frog (*Silurana (Xenopus) tropicalis*), zebrafish (*Danio rerio*), medaka (*Oryzias latipes*), fugu (*Takifugu rubripes*), three-spined stickleback (*Gasterosteus aculeatus*), green spotted pufferfish (*Tetraodon nigroviridis*) and sea lamprey (*Petromyzon marinus*). In order to appropriately root the phylogenetic trees, corresponding searches were made in the Ensembl genome databases for a tunicate (*Ciona intestinalis*) and a nematode (*Caenorhabditis elegans*) or fruitfly (*Drosophila melanogaster*), as well as in the lancelet (*Branchiostoma floridae)* in the National Center for Biotechnology Information (NCBI) databases at http://www.ncbi.nlm.nih.gov. In Ensembl, the protein predictions representing the best BLAST hits were collected and their chromosome locations were noted. For short, incomplete or divergent protein predictions, better predictions were manually curated from the corresponding genomic sequence with regard to consensus start and stop codons, splice donor and acceptor sites and sequence similarity to other identified family members. Expressed sequence tags (ESTs) curated and aligned by the Ensembl database were also considered. The InterPro database of protein domain predictions (www.ebi.ac.uk/interpro) was used to identify conserved protein domains. Ensembl searches were initiated in database versions 55 (July 2009) and 56 (September 2009), and simultaneously in the *pre.ensembl.org* database for the sea lamprey genome. All sequences and database identifiers were verified against the most updated genome assembly versions as shown in Ensemble database version 66 (February 2012). This information can be found in Table S1.

To identify putative paralemmin family members in the lancelet genome with greater certainty, a protein blast search was performed using the pattern hit initiated algorithm (PHI-BLAST) in the NCBI non-redundant protein sequence database. The identified zebrafish palmdelphin-B (see Results) was used as query and the conserved amino acid motif KX[KR]XXR[ED]XWL[ML], identified from a preliminary alignment of the identified vertebrate paralemmin homologs, was entered as PHI-pattern.

### Identification of neighboring gene families

Protein families, as defined by the automatic Ensembl database protein family predictions, which had members closer than 5 Mb to at least three different paralemmins in the human genome were considered for synteny analyses. The protein sequences corresponding to the best gene predictions for these families were collected and their chromosomal locations noted. These sequences were used for tblastn searches in order to identify additional family members. The *AKAP2* gene and its protein family members were also included in the analysis since *AKAP2* has been reported to be transcribed together with paralemmin-2 [10]. These protein families were analyzed as described for the paralemmins with regard to species representation (except green spotted pufferfish), sequence alignment and phylogenetic analyses.

### Sequence alignment and phylogenetic analyses

The identified protein predictions from the database searches were used to produce amino acid alignments using the ClustalW [29] tool with stardard settings in Jalview2.4 [30]. Green spotted pufferfish sequences were included in the phylogenetic analyses of the paralemmin gene family, but not of neighboring gene families due to the close evolutionary relatedness of this species and fugu. The final alignments were manually inspected using Jalview with regard to incomplete protein sequence predictions and poorly aligned sequence stretches. Details on the sequence curation and alignment editing process can be given upon request.

Two bootstrapped phylogenetic methods were applied on the alignments: a neighbor joining (NJ) analysis [31] and a phylogenetic maximum likelihood (PhyML) analysis [32]. The NJ tree construction method (with 1000 bootstrap replicates) was applied with standard settings (Gonnet weight matrix, gap opening penalty 10.0 and gap extension penalty 0.20) in ClustalX 2.0 [29]. The PhyML method was applied using the web-application of the PhyML 3.0 algorithm available at http://www.atgc-montpellier.fr/phyml/ with the following settings: amino acid frequencies (equilibrium frequencies), proportion of invariable sites and gamma-shape parameters were estimated from the datasets; the number of substitution rate categories was set to 8; BIONJ was chosen to create the starting tree and both the NNI and SPR tree improvement methods were used to estimate the best topology; both tree topology and branch length optimization were chosen. For branch support a bootstrap analysis with 100 replicates was chosen. The best amino acid substitution models for the PhyML analyses were estimated from the alignments using ProtTest 1.4 [33]. Models were tested with no add-ons and assuming eight gamma rate categories, the optimization strategy was set to slow and the BIONJ strategy was selected for the random input tree. The JTT model was assumed for all PhyML analyses based on the ProtTest results.

The paralemmin gene family tree was not rooted since no complete invertebrate paralemmin sequence could be identified. Regarding the neighboring gene families, identified nematode sequences were used if such a sequence could be found, if not, identified lancelet sequences were used. If no invertebrate protein prediction could be found, the trees were unrooted.

### EST sequence analysis of read-through transcripts

To detect possible read-through transcripts similar to the Palm2-AKAP2, the chromosome locations of all identified paralemmin family members were analysed with regard to the expressed sequence tags (ESTs) that are automatically identified and aligned to the genomic sequence by the Ensembl database. These EST sequences are gathered from the NCBI dbEST database at http://www.ncbi.nlm.nih.gov/dbEST.

## Results

### Sequence identification of paralemmins

The BLAST searches of the genome databases, using the amino acid sequences of the known paralemmins in human and mouse (*PALM1*, *-2*, *-3*, and *PALMD*) as queries enabled us to identify paralemmin sequences in several other vertebrates. In tetrapods, aside from human and mouse, the chicken and Western clawed frog genomes were searched. We could identify *PALM1*, *PALM2* and *PALMD* sequences in all four genomes, while a *PALM3* sequence could only be identified in the human, mouse and Western clawed frog genomes. The lack of a *PALM3* sequence in the chicken genome could be due to a genome sequencing or assembly error. Therefore we also searched the zebra finch (*Taeniopy*gia guttata), duck (*Anas platyrhynchos*) and turkey (*Meleagris gallopavo*) genomes, but no *PALM3* sequence could be identified in these bird genomes either, indicating that *PALM3* has been lost from the bird lineage.

**Figure 1:**
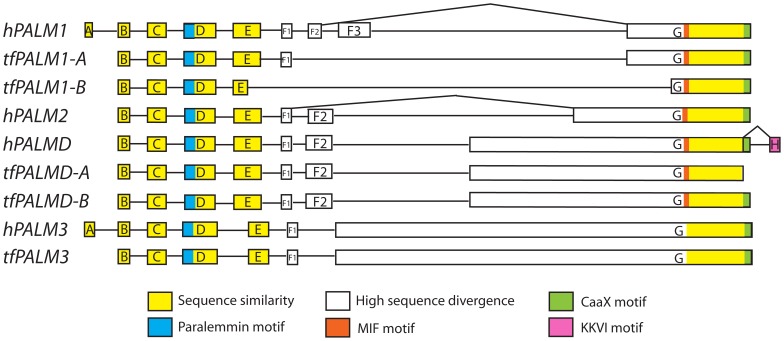
Conserved organization of paralemmin isoform genes. Exon sizes are roughly proportional relative to each other, whereas intron sizes are schematic. Kinked lines indicate differential splicing. Human (h-) and teleost fish (tf-) genes are shown as examples.

In the teleost fish genomes up to six different paralemmin sequences could be identified, including *PALM2, PALM3* and duplicates of *PALM1* and *PALMD*. However, there appear to be several differential losses of paralemmin genes in the teleost genomes. We have named the teleost-specific duplicates paralemmin-1A and -B (*PALM1-A* and –*B*) and palmdelphin-A and –B (*PALMD-A* and –*B*) because their chromosomal locations suggest origin in 3R (described below), and such duplicates are usually designated with A and B. The greatest diversity could be found in the zebrafish genome with six paralemmin genes. The identified paralemmin genes and their chromosome locations are shown in Table 1. The accession numbers of all identified gene predictions can be found in Table S1. A partial *PALM1-A*-like sequence could be identified in the green spotted pufferfish including only exon F3 and a short segment of exon G (see Figure 1), similarly only a single partial *PALM3*–like sequence could be identified in medaka. Due to genome sequencing or assembly errors in these genome assemblies, these sequences could not be predicted in their entirety and were therefore not included in the phylogenetic analyses.

**Table 1:** Identified *PALM* genes and their chromosomal locations.

	*PALM1*	*PALM2*	*PALM3*	*PALMD*
Human (Hsa)	19: 0.71^a^	9: 112.54^a^ ^c^	19: 14.17	1: 100.11
Mouse (Mmu)	10: 79.26^a^	4: 57.58^a^ ^c^	8: 86.55	3: 116.67
Chicken (Gga)	28: 2.25^a^	Z: 64.23^a^	-	8: 12.87^e^
Western clawed frog (Xtr)	s_289: 1.39^a^	s_229: 1.52^a^	s_649: 0.15	s_252: 0.88

	*PALM1-A*	*PALM1-B*	*PALM2*	*PALM3*	*PALMD-A*	*PALMD-B*
Zebrafish (Dre)	11: 14.44^a^	2: 26.38	10: 4.91^a^	6:9.59^a^	24: 30.85^a^ ^d^	2: 18.92^a^
Medaka (Ola)	- a	22: 4.42	12: 18.32^a^	s6823:1b^a^ ^e^	u_236: 1.11^a^	- a
Fugu (Tru)	s_269:0.17^a^	s_158: 0.27	s_409: 0.14^a^	s207:0.31^a^	-	s_285:0.14^a^ ^b^
Stickleback (Gac)	VIII: 17.39^a^	-	XIV: 9.36^a^	IX: 17.53^a^	s_95:0.36^a^	III: 5.24^a^ ^b^
Green spotted pufferfish (Tni)	s_10234:14.32^e^	10: 9.63	4: 0.98^a^	18:4.82^a^	-	s_7729: 58.61^a^ ^b^
Lamprey (Pma)	GL477367: 9.36 Kb					
Lancelet (Bfl)	scaffold 302					

a *PALM* genes with an identified adjacent *AKAP2* homolog/paralemmin downstream gene (PDG). For a detailed list of PDG gene locations see Table S1. In the case of Medaka, no *PALM1-A or PALMD-B* gene was found, but *PDG1A* and *PDG4B* genes could be predicted in the expected respective locations. *PDG3B* genes were found in stickleback and in medaka but are not listed in this table.

b The identified *PDG* genes are located on the opposite strand to these *PALMD-B* genes.

c Known Palm2-AKAP2 fusion protein [10].

d Read-through ESTs of *PALMD-A* and *PDG4B* detected. See Results.

e The identified partial *PALM1-A* like sequence in Green spotted pufferfish and partial *PALM3* sequence in medaka were not used in the phylogenetic analyses. See Results.

Chromosome assignments are given where possible, “s_” denotes assignment to unassembled scaffolds and “u_” denotes assignment to unassembled ultracontigs. Locations are given in megabases, unless specified. Information about genomic assembly versions can be found in Table S1.

Partial sequences of at least three different paralemmin genes were found in the elephant shark (*Callorhinchus milii*) genome database, but since they were found on very short genomic scaffolds and covered different regions of the proteins, they could not be included in alignments for phylogenetic analyses (data not shown). More importantly, one paralemmin sequence could be found in the jawless vertebrate genome of sea lamprey, *Petromyzon marinus*, representing a lineage that is known to have diverged before the origin of jawed vertebrates.

When all the identified vertebrate paralemmin sequences were aligned, a conserved motif in the N-terminal region became apparent (see below). A pattern-hit initiated Blast (PHI-blast) search with this motif (KX[KR]XXR[ED]XWL[ML]) in the lancelet generated one hit on genomic scaffold 302 (assembly v1.0) and a putative exon containing this motif could be predicted (see Discussion). The Blast-hit can be accessed in the *Branchiostoma floridae* v1.0 browser available at http://genome.jgi-psf.org/ (last accessed May 24, 2012) in the region scaffold_302:250750-250600 (minus strand). Positive hits for paralemmin sequences could not be found in any other invertebrate genome such as the extensively analyzed genomes of the tunicate *Ciona intestinalis*, the sea urchin *Strongylocentrotus purpurat*us, the fruitfly *Drosophila melanogaster*, and the nematode *Caenorhabditis elegans*.

### Paralemmin gene organization and sequence features

**Figure 2:**
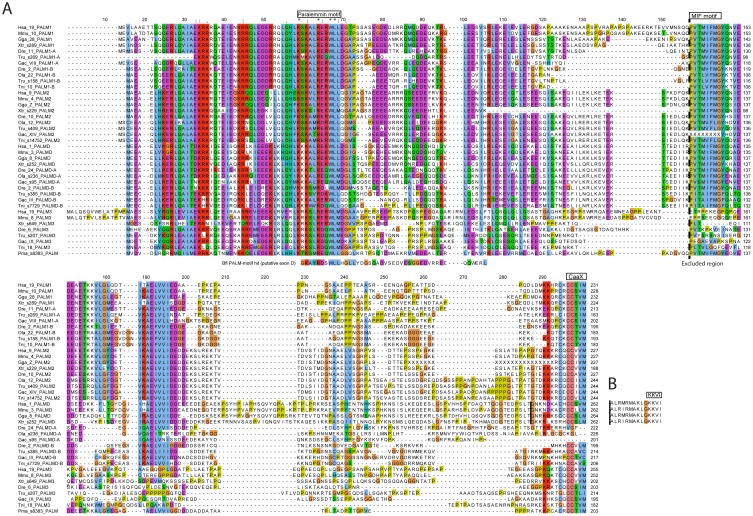
Paralemmin sequence alignments. A) Alignment of the conserved sequence regions of paralemmin orthologs and paralogs. These sequence regions are colored in Figure 1 (exons F1–F3 and 5′ parts of exon G are excluded). The point where the less conserved region is excluded from the alignment is marked by a dashed vertical line at position 158. Asterisks mark invariant residues and dots mark conservative substitutions. The paralemmin motif (conserved in all paralemmins), the MIF motif (conserved in all but *PALM3*) and the C-terminal CaaX motif are indicated by boxes above the alignment. The alternative splice site of *PALMD* genes is indicated by a black vertical line at position 288. B) The alternatively spliced exon H in *PALMD* genes, with the KKVI motif marked. The alignment used in the phylogenetic analyses included the *PALMD* splice variants with exon H, including the KKVI motif, rather than the splice variants with the CaaX motif (see Results). Species name abbreviations: human (Hsa), mouse (Mmu), chicken (Gga), Western clawed frog (Xtr), zebrafish (Dre), medaka (Ola), fugu (Tru), three-spined stickleback (Gac), green spotted pufferfish (Tni), sea lamprey (Pma) and lancelet (Bfl). Chromosome, scaffold (s-) or ultracontig (u-) locations are given in the sequence names, followed by the assigned paralemmin isoform based on our phylogenetic analysis.

The identification and annotation of paralemmin genes in the different vertebrate genomes revealed that they have a common gene organization (Figure 1). The 5′ parts of the genes contain six to eight small exons (A-F3) that are followed by a large 3′ exon (G). This mirrors the previously known gene organization of *PALM1*, *PALM2* and *PALMD* in human and mouse [10]. Still, paralemmin sequences diverge substantially between species and isoforms. For instance, the amino acid sequence of paralemmin-3 is almost twice as long as that of paralemmin-1, a difference that is largely due to an earlier start for the last large exon of *PALM3* (exon G in Figure 1). The corresponding exon of *PALMD* also starts earlier than in *PALM1* and *PALM2*. Because of these differences, it was important to identify conserved sequence features for all identified paralemmin genes in order be able to use the relevant sequence regions for the phylogenetic analysis. We found regions of sequence similarity in the first exons (A–E) and the end of the last exon (G), interrupted by a region of high sequence divergence (Fig. 1). The regions of sequence similarity (colored in Fig. 1) were used to generate the alignment in Figure 2A for the phylogenetic analyses. The alignment data file is available upon request.

Within the regions of sequence similarity, several conserved amino acid sequence motifs became apparent. Only one such motif is conserved in all identified paralemmin sequences, why we have chosen to call it the paralemmin motif. This motif encoded in exon D consists of 11 highly conserved residues of which four are invariant and three represent conservative substitutions in the species we have investigated (Fig. 2A). This sequence motif was used to search for invertebrate paralemmins as mentioned above. The known tripeptide methioninine-isoleucine-phenylalanine (MIF) conserved in human and mouse *PALM1*, *PALM2* and *PALMD* [10] could be identified as part of a larger stretch of 7 conserved residues in all paralemmin sequences except *PALM3*, the lancelet homolog and stickleback *PALM2* (Fig. 2A). The M and I residues are not conserved across all sequences while the F and the following G residue are completely conserved in our alignment. This stretch, encoded by exon G, has been named the MIF motif [10]. The C-terminal CaaX motif could also be identified in all sequences except teleost *PALMD-A* sequences (Figure 2A). In fact, the teleost PALMD-A sequences show no apparent sequence conservation between them in the C- terminal. The alternatively spliced C-terminal KKVI motif could be identified in all tetrapod *PALMD* sequences, but not in any teleost *PALMD*s (Fig. 2B). This KKVI motif is encoded by an additional downstream exon that we call exon H. The amino acid sequence encoded by this exon is highly conserved in all identified *PALMD* sequences; only one out of 14 positions deviates (Fig. 2B).

*PALMD* has two splice variants; one contains the CaaX motif and the other uses exon H with the KKVI motif. In the alignment used for the phylogenetic analyses, the splice variant with the CaaX motif was used due to higher sequence conservation within the *PALM genes*, even though the cytosolic *PALMD* splice variant without the CaaX is the more frequent variant. The alignments in Figure 2 show both splice variants.

### Phylogenetic analysis of the paralemmin gene family

**Figure 3:**
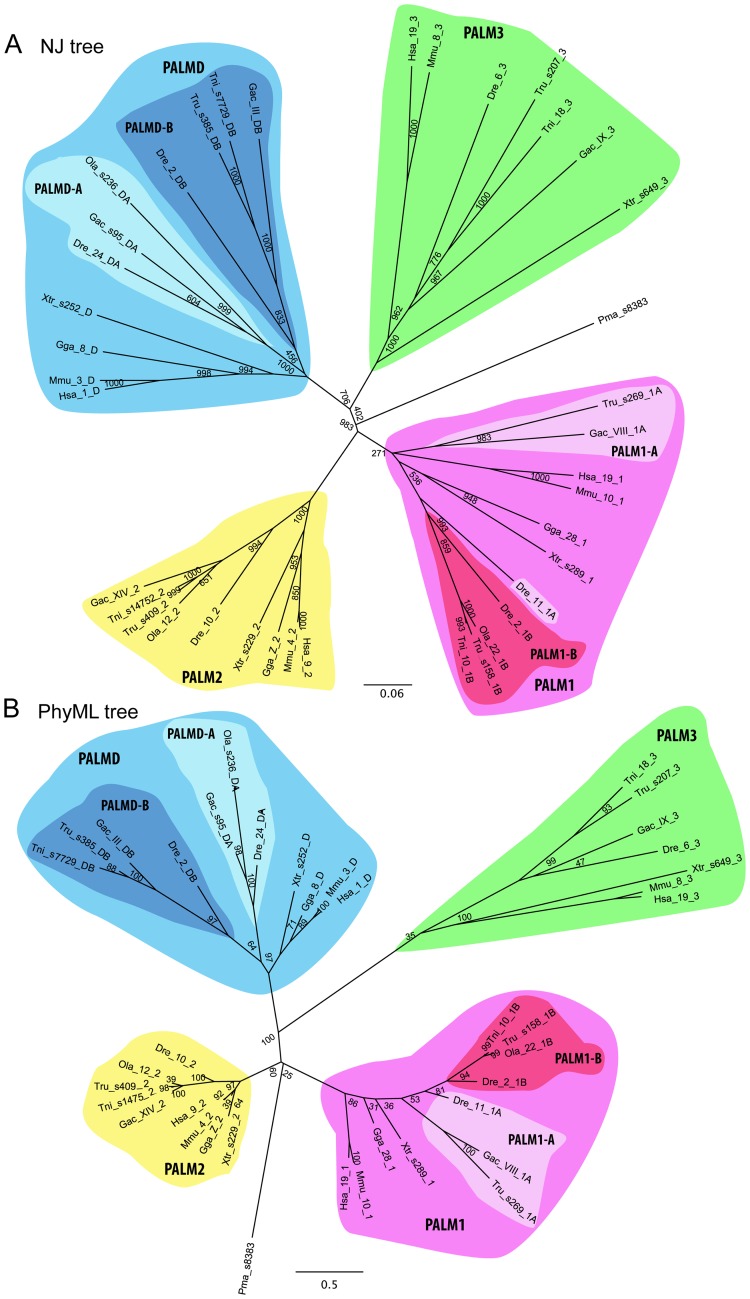
Phylogenetic analyses of the paralemmin protein family. A) Bootstrapped neighbor-joining tree (1000 bootstrap replicates). B) Bootstrapped phylogenetic maximum likelihood tree (100 bootstrap replicates). Species name abbreviations are applied as in Figure 2. The chromosome, scaffold (s-) or ultracontig (u-) assignments are given in the sequence names after the species abbreviations. Both trees are presented as radial unrooted trees since no invertebrate orthologs could be identified to be used as outgroup.

Our phylogenetic analyses of the paralemmin sequences using both the distance-based neighbor joining method (NJ) and the phylogenetic maximum likelihood method (PhyML) concurrently show that the paralemmins form four distinct clusters: *PALM1*, *PALM2*, *PALM3* and *PALMD* (Figure 3). The *PALM2* and *PALMD* clusters are well supported in both analyses, however, the lower bootstrap support for the *PALM1* branch in the NJ tree and the *PALM3* branch in the PhyML tree likely reflect a larger degree of sequence divergence within these clusters. Notably, the internal topology of the *PALM1* cluster could not be resolved with high bootstrap support using either method, indicating a larger degree of divergence between the *PALM1* sequences in the different species. The teleost-specific duplicates *PALMD-A* and *PALMD-B* form well-supported branches within the *PALMD* cluster with both phylogenetic methods. With the topology within the *PALM1* cluster being less resolved, there is not a clear teleost-specific *PALM1-A* and *PALM1-B* divergence, although the *PALM1-B* sequences cluster together with high bootstrap support in both trees (Figure 3). The identified lamprey sequence could not be assigned to a specific subtype in either analysis, although it seems to be more similar to *PALM1* and *PALM2*.

The phylogenetic trees in Figure 3 show that *PALM3* is the least conserved of the paralemmin isoforms, as indicated by the longer branch-lengths within this cluster. Sequence conservation between the species included in the analysis and with the other paralemmin isoforms is low, only showing high identity within the paralemmin and CaaX motifs (Figure 2). The MIF motif described above could not be identified in the *PALM3* sequences.

### Analysis of neighboring gene families and identification of conserved synteny blocks

**Figure 4:**
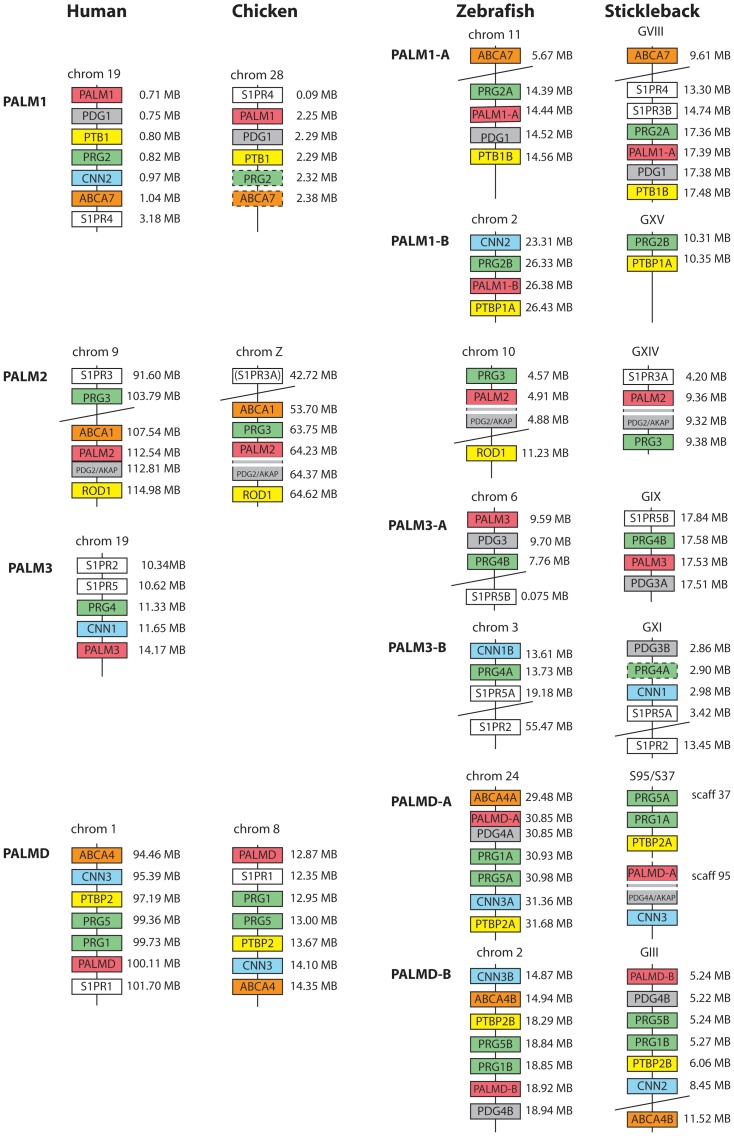
Identified blocks of conserved synteny around paralemmin genes in human, chicken, zebrafish and stickleback. Gene families that were selected had members located within ±5 Mb of at least three different paralemmin genes in the human genome. Genes in boxes with dashed frame were not included in the phylogenetic analyses (see Results). Gene family abbreviations: ATP-binding cassette sub-family A (ABCA), calponin (CNN), paralemmin downstream genes (PDG), plasticity related genes (PRG), polypyrimidine tract-binding protein (PTBP), sphingosine-1–phosphate receptors (S1PR). These gene families have additional members on separate chromosomes, not part of these identified blocks of conserved synteny (Table S1, Figure 5, Figures S1, S2, S3, S4, and S5); for the S1PR family these are *S1PR1*, *S1PR3* and *S1PR4* on zebrafish chromosome 22 and *S1PR1* on stickleback group VIII; for the CNN family, *CNN1A* is located on chromosome 1 in zebrafish; for the ABCA family, *ABCA1* in zebrafish is located on chromosome 1 and *ABCA4A* in stickleback is located on group XXI.

**Figure 5:**
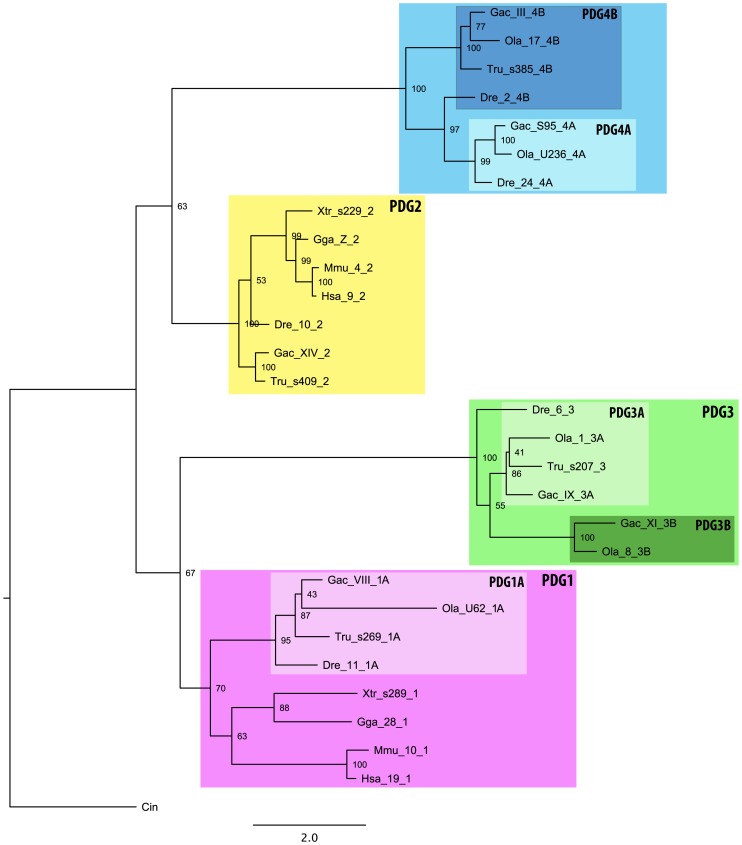
Phylogenetic maximum likelihood tree of the paralemmin downstream gene (PDG) family. This family includes all identified homologs of the known *AKAP2* genes. The *PDG* isoforms are named for the paralemmin genes to which they are adjacent, with the exception of *PDG4*, which are adjacent to *PALMD* genes. The phylogenetic analysis of this family, as well as the chromosomal data, are consistent with our proposed duplication scheme (Figure 6) and the phylogenetic analysis of the paralemmins (Figure 3), but also show a duplication of the region bearing *PALM3* in teleost fishes, probably through 3R.

To investigate whether the paralemmin gene family expanded through the early vertebrate genome duplications, and if the additional teleost duplicates are the result of the 3R event, we identified gene families with members adjacent to the paralemmin genes and performed phylogenetic analyses. The identified families are; sphingosine-1–phosphate receptors (*S1PR*), plasticity related genes (*PRG*) (also called lipid phosphate phosphatase related proteins), ATP-binding cassette sub-family A (*ABCA*), calponin (*CNN*), and polypyrimidine tract-binding protein (*PTBP*). This selection of neighboring families represents all Ensembl protein family predictions with members closer than 5 Mb to at least three paralemmin genes in the human genome. Additionally, homologs of the sometimes co-transcribed AKAP2 were identified by sequence homology and chromosomal location adjacent to a paralemmin gene (see below). The identified blocks of conserved synteny around the paralemmin genes in human, chicken, zebrafish and stickleback are shown in Figure 4. Phylogenetic trees using the PhyML method were made for each protein family (Figure 5, Figures S1, S2, S3, S4, and S5), which allowed us to assign new sequence predictions their correct subtype names within the families. The accession IDs of the identified Ensembl protein family predictions as well as all identified family members are shown in Table S1. The assigned sequence names of newly predicted family members are written in a separate column. Three identified sequences were not included in the phylogenetic analyses because of gaps in the genomic read or spurious regions of poor sequence alignment: PRG2 and ABCA7 in chicken, and PRG4A in stickleback. These boxes are shown in Figure 4 with a dashed frame.

### Identification of paralemmin downstream gene family (PDG)

The gene located 3′ to *PALM2*, *AKAP2*, has been reported to give rise to a fusion protein (Palm2-AKAP2). Therefore we were interested in identifying homologs of this gene downstream of other paralemmin genes in the studied genomes. We found genes downstream of *PALM1* that have sequence similarity to *AKAP2* in most studied genomes (Table 1). In teleost fish genomes, *AKAP2* homologs could only be found downstream of *PALM1-A* but not *PALM1-B* genes. In the green spotted pufferfish genome it was not possible to find homologs of *AKAP2* downstream of either *PALM1-A* or *-B*. Furthermore, we identified *AKAP2* homologs downstream of *PALM3, PALMD-A* and *PALMD-B* in the teleost genomes, but not downstream of tetrapod *PALM3* and *PALMD* genes. No *AKAP2* homologs could be identified in the sea lamprey or elephant shark genomes.

We have called this family of *AKAP2* homologs, paralemmin downstream genes (*PDG*). Our phylogenetic analysis of *PDG* sequences shows four well-supported clades corresponding to the chromosomal locations adjacent to *PALM1*, *PALM2, PALM3* and *PALMD* genes (Figure 5); therefore we have assigned the names *PDG1*, *PDG2* (*AKAP2*), *PDG3* and *PDG4* (located downstream of palmdelphin, the fourth paralemmin family member) to the identified sequences. The *PDG* genes, including *AKAP2*, are summarized in Table 1 and their chromosome locations are shown in Table S1. The amino acid alignment of the *PDG* sequences is shown in Figure S6. Although there is significant sequence conservation between species and between the duplicates, we could only identify one region with reasonably high conservation in the C-terminal, starting around position 1290 of the alignment (Figure S6). This region includes two invariant positions (WE) as well as two positions representing conservative substitutions across all homologs in all studied species, with additional positions showing isoform-specific conservation. We have marked the small motif of 11 amino acids encompassing the most conserved positions as the PDG motif in Figure S6. Additionally there is a region around positions 1127–1160 rich in positively and negatively charged amino acid residues showing a high degree of isoform-specific conservation.

To investigate if the other *PDG*s are co-transcribed with their upstream paralemmin gene, we analyzed the chromosomal locations with regard to the expressed sequence tags (ESTs) automatically aligned by the Ensembl database (see Methods). However, no tetrapod fusion ESTs could be identified in the database except for the known fusions with *PALM2*. In teleost fishes we found three read-through ESTs in zebrafish that suggest the existence of a fusion protein of *PALMD-A* and its downstream *PDG4A*: EB950131, EB946765 and EB950439. No other fusion ESTs were found in teleosts, not even homologs to the known Palm2-AKAP2. The second teleost-specific *PDG* gene, *PDG4B*, is found in all studied teleost genomes, but except for zebrafish it is encoded on the opposite strand to *PALMD-B*. These findings are summarized in Table 1.

## Discussion

### Evolution of the paralemmin gene family

The paralemmin family of proteins consists of four members encoded by four different genes, *PALM1*, -*2*, -*3* and *PALMD*, in the investigated mammalian genomes and in the genome of the frog *Xenopus tropicalis*. It consists of three members in the chicken genome as the *PALM3* gene appears to have been lost from the avian lineage. These results are summarized in Table 1. Due to considerable sequence divergence between the four isoforms, and the absence of invertebrate orthologs, their evolutionary history was difficult to deduce solely based upon sequence information and sequence-based phylogenetic analyses. In previous studies of other gene families it has been possible to resolve the evolution by combining sequence data with information on the chromosomal locations of the genes across a wide selection of vertebrate genomes. This includes investigating the conserved synteny of gene families adjacent to the *PALM* genes as well as the possible paralogy relationships between the chromosome segments. Thereby one can also use the phylogenetic analyses and chromosomal data of the adjacent gene families to see if these share the same evolutionary history and may perhaps give a clearer picture of the events.

By analyzing both phylogenetic trees and chromosomal data, we conclude that the four tetrapod paralemmins arose from a single ancestral gene in the two basal vertebrate tetraploidizations, 2R, prior to the origin of jawed vertebrates. Although our phylogenetic analyses of the paralemmins are unrooted (Figure 3) because no true invertebrate ortholog could be identified, the time-frame for the divergence of the four paralemmin isotype clusters is shown by the rooted phylogenetic analyses of the neighboring gene families, which support the divergence of the four vertebrate paralemmin lineages or clades in the same time-frame as 2R: before the divergence of lobe-finned fish (including tetrapods) and ray-finned fish, and after the emergence of vertebrates.

Our discovery of a sequence fragment with paralemmin features in the lancelet was surprising because paralemmins are considered vertebrate specific [1], [34]. While this finding potentially supports the presence of an ancestral *PALM* gene in the common ancestor of the lancelet and vertebrates, the identified sequence only constitutes a putative prediction of exon D (Figure 1) with sequence similarity to the identified paralemmin-motif and sheds little light on the structure of paralemmin before the emergence of vertebrates. The identified sequence can be seen in Figure 2A aligned slightly below the rest of the alignment. The sequence includes most of the paralemmin motif, with three of the four invariant positions and all three positions showing conservative substitutions. The fourth invariant position is on another exon in the other species.

It is not possible to determine the orthology relationships of the single paralemmin sequence that was found in the genome of the sea lamprey *Petromyzon marinus*. In the phylogenetic maximum likelihood tree there is not enough phylogenetic resolution to confidently assign it to a paralemmin subtype (bootstrap value 25), although it seems to be more similar to *PALM1* and *PALM2* (Figure 3B), while the neighbor joining tree suggests it represents an ancestral lineage including both *PALM1* and *PALM2* (Figure 3A) with somewhat higher support. It is not clear whether the lineage leading to the sea lamprey underwent the 2R tetraploidizations or possibly only one of them [35], [36].

Interestingly, none of the neighboring families in the blocks of conserved synteny around *PALM3* have orthologs in the bird genomes that were investigated (Figure 5, Figures S1, S2, S3, S4, and S5, summarized in Figure 4), indicating that *PALM3* was lost as part of a larger block in this lineage. Our phylogenetic analyses (Figure 3) also suggest that the tetrapod *PALM3* sequences evolve more rapidly than the other isoform genes, indicating a lower conservative selection pressure. The larger distances between the sequences in the tree, point towards an increased rate of substitutions in this lineage.

In addition to the expansion in 2R, the teleost-specific tetraploidization, 3R, produced additional isoforms of *PALM1* and *PALMD* (Table 1, Figure 3). These duplicates, which we have called *PALM1-A* and –*B* and *PALMD-A* and –*B*, could all be found in the zebrafish and medaka genomes. In the stickleback, fugu and green-spotted pufferfish genomes there appear to have been differential losses (Table 1, Figure 3).

The support for the origin of the four paralemmin isoform genes by the early vertebrate whole genome duplications, and the subsequent expansions in the teleost-specific tetraploidization, comes from the analysis of conserved synteny blocks around the *PALM* genes (Figure 4) as well as the phylogenetic analyses of their chromosomal neighbors (Figure 5, Figures S1, S2, S3, S4, and S5). Our synteny analyses of the regions spanning 5 Mb around each paralemmin gene identified six additional gene families that seemed to belong to the same paralogon, i.e. that quadruplicated as part of the same chromosomal block as the ancestral paralemmin gene and would thus share the same evolutionary history. The phylogenetic analyses of these gene families, using identified lancelet, tunicate or nematode *Caenorhabditis elegans* sequences as outgroup, are shown in Figure 5 and Figures S1, S2, S3, S4, and S5. The phylogenetic trees of the ATP-binding cassette subfamily A (ABCA), calponin (CNN), polypyrimidine tract-binding protein (PTBP) and paralemmin downstream gene (PDG) families are consistent with the phylogenetic analyses of the paralemmins (Figure 3), and support their concomitant duplications as part of the same chromosome block. However, there have been differential gene losses: The CNN family lacks paralogs neighboring *PALM2* genes, while the ABCA and PTPB families lack paralogs neighboring *PALM3* genes (Figures S1, S2, S3, S4, and S5). The phylogenies of the sphingosine-1-phosphatase related proteins (S1PR) and plasticity related genes (PRG) families are more difficult to resolve. The phylogenetic tree of the PRG family is not fully consistent with the phylogenetic analysis of the paralemmins (Figure S4). However, this is likely due to a local gene duplication before the basal tetraploidizations. The PRG family has genes neighboring all four paralemmin isoform genes in all of the investigated genomes, and their chromosomal locations are consistent with the expansion of the family as part of the same chromosome block as the *PALM* genes (Figure 4, Table S1). As for the S1PR family, there appears to have been a local duplication after the basal tetraploidization as well as several translocations in the teleost lineage. Additionally, no homolog could be identified in the investigated invertebrate genomes, which makes the relative dating of the duplication events difficult (Figure S3). However, there are homologs neighboring all four paralemmin isoform genes in all tetrapod genomes, except that of the Western clawed frog where mapping data is not available, which indicates that the family expanded in the same time-frame and chromosomal block as the *PALM* genes (Table S1), but that secondary events complicate the tree topology (Figure S3).

**Figure 6:**
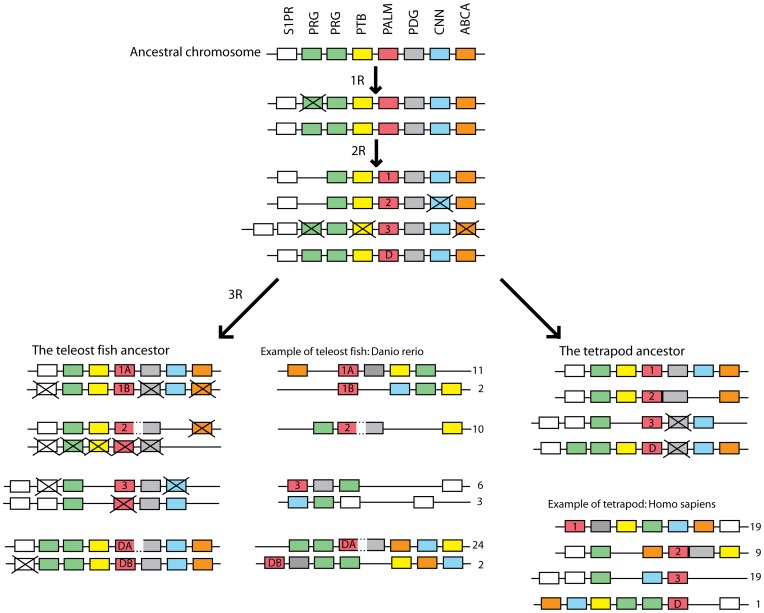
Proposed scenario for the evolution of the paralemmin gene family. A single ancestral chromosome was quadrupled in the two basal vertebrate rounds of genome doubling (1R and 2R), giving rise to the four paralemmin isoform genes *PALM1, PALM2, PALM3* and *PALMD*. The *PALM3* gene appears to have been lost from the avian lineage (not shown here). Subsequently, the teleost-specific third round of genome doubling (3R), generated duplicates of *PALM1* and *PALMD*. This duplication scheme is supported by the chromosome locations and phylogenetic analyses of the PALM gene family as well as the neighboring gene families ABCA, CNN, PDG, PTB, PRG and S1PR across a wide selection of vertebrate species. Here zebrafish (*Danio rerio*) and human are shown as examples. Note that two of the duplicated genome regions in zebrafish have ended up on chromosome 2, likely due to chromosome rearrangements in the zebrafish lineage. Similarly, two of the chromosome regions in the human genome harboring *PALM1* and *PALM3*, respectively, are on different parts of chromosome 19. However, this seems to be due to a recent fusion in the linage leading to humans, as detailed in the Discussion. Note also that several genes have been lost after the chromosome duplications and that the gene order has been shuffled in both zebrafish and human compared to the predicted ancestral chromosome regions. Crossed-over boxes represent likely gene losses. Dotted lines between *PALM2* and PDG genes indicate read-through transcription and splicing into the same mRNA. Gene family abbreviations and colors are applied as in Figure 4 with the PALM gene family in red.

Together with the synteny analyses (summarized in Figure 4), these phylogenetic data support duplication of these chromosomal regions in the basal tetraploidizations or are at least consistent with such a scenario. The neighboring gene families also support further duplications in the teleost 3R event, as detailed in the supporting information. Our proposed evolutionary scenario is presented in Figure 6.

The chromosomal regions that harbor the *PALM* genes have previously been identified as belonging to a paralogy group, i.e. regions that are derived from the two early vertebrate rounds of tetraploidization, 2R. In the analysis of the lancelet genome and the evolution of the vertebrate karyotype [37] the *PALM*-bearing segments of human chromosomes 1 (91.24–102.06 Mb), 9 (93.15–114.84 Mb) and two segments of chromosome 19 (1–9.86 Mb and 9.86–15.44 Mb) were found to represent four ancient blocks of conserved linkage. These segments were also found to correspond to the same ancestral chromosome before 2R in a reconstruction of the ancestral vertebrate genome and evolution of vertebrate chromosomes [13]. The two segments of chromosome 19, which harbor *PALM1*, and *PALM3*, respectively, likely represent a fusion event in the lineage leading to humans. In the mouse genome the *PALM1* and *PALM3* genes are located on different chromosomes, 10 and 8 respectively (Table 1). This is consistent with a large-scale rearrangement of synteny regions between the mouse and human genomes (see for instance the homology map at http://www.ncbi.nlm.nih.gov/projects/homology/maps/human/chr19/, *last accessed May 23, 2012*).

After the divergence of the paralemmin gene family, the paralemmin isoform genes also evolved differential expression profiles. In mice, palmdelphin protein is broadly expressed in virtually all tissues analyzed, with highest expression in the heart [1], whereas the paralemmin-1 protein and mRNA are detected in most tissues but at much more differential levels than palmdelphin, and by far highest expreeion in the brain [4]. Paralemmin-2, Palm2-AKAP2 and paralemmin-3 have yet other patterns of tissue distribution (GH and MWK,unpublished data). A feature shared by all four genes, however, is that the nervous system is among the tissues in which they are most highly expressed.

### Sequence features of the paralemmin proteins

Detailed inspection of all paralemmin sequences allowed us to identify a few well-conserved sequence motifs. Exon D encodes a stretch of 11 highly conserved residues, of which four are invariant and three represent conservative substitutions, which we have called the paralemmin motif (Figures 1 and 2). Because this domain is so well conserved across species and paralemmin isoforms it is likely to be associated with a conserved function. Other highly conserved features in the paralemmins are the MIF motif encoded by exon G and the CaaX/KKVI motifs at the C-terminus. The MIF motif consists of 7 residues that show a high degree of conservation in all paralemmins except paralemmin-3 and surprisingly the stickleback paralemmin-2 (Figure 2). The paralemmin-3 sequences differ between species in this stretch, indicating absence of conservative selection pressure. However, occasional sequence similarity between species downstream of this point suggests that the paralemmin-3 sequences have changed by amino acid substitution rather than insertion or deletion.

The CaaX motif that attaches the paralemmins to the cell membrane by a lipid anchor is conserved in all paralemmins except palmdelphin-A in teleosts. We have not found any conserved C-terminal motif for palmdelphin-A; it has neither the CaaX nor the KKVI motif, whereas in palmdelphin-B we identified the CaaX motif (Figure 2). This shows that the loss of the palmdelphin-A CaaX motif happened after the teleost 3R event. In mammals, the single palmdelphin gene can give rise to either a membrane-anchored or a cytosolic variant of palmdelphin through two different splice variants using either the CaaX membrane-anchoring motif or the conserved KKVI motif encoded by exon H (Figures 1 and 2). The cytosolic variant is the predominant expression product of mammalian palmdelphin [10]. Based on the differing sequence features, it is possible that in teleost fish this specialization is accounted for by two separate proteins; one of the 3R duplicates, palmdelphin-A, is the cytosolic form, even though they seem to lack the alternatively spliced exon H, while the other, palmdelphin-B, is the membrane-anchored version of the protein. The overall identity of zebrafish palmdelphin-A and palmdelphin-B is 41%, suggesting that perhaps also other functions have become subdivided between the two gene products. The conserved KKVI motif in the tetrapod palmdelphins is also preceded by an extended highly conserved stretch, with 13 out of the last 14 amino acids between human, mouse, chicken and frog identical, indicating functional importance perhaps for the binding of glutamine synthetase [1].

The C-terminus of palmdelphin-B in teleost fish has only two cysteines among the last 7 amino acids while all the other family members with CaaX boxes have three (Figure 2). The most C-terminal cysteine is prenylated and the preceding cysteines are palmitoylated to constitute a membrane anchor. It has been reported that mutagenesis of any of these three cysteines in paralemmin-1 caused the protein to lose its ability to increase the number of filopodia and spines in transfected cells [2]. Therefore, if palmdelphin-B has lost one lipidation site, it may be expected to have acquired different membrane interaction properties than the family members that do have three cysteines.

### The paralemmin downstream genes (PDG)

It was previously reported that the *AKAP2* gene immediately downstream of *PALM2* in human, mouse and rat can be co-transcribed with *PALM2* to generate a fusion protein [10], [38]. This opens an intriguing possibility regarding co-transcription in other species as well as for the other paralemmins. We searched for *AKAP2*-related genes downstream of all identified paralemmin family genes in all species' genomes and constructed a sequence-based phylogenetic tree of this gene family, which we have called the paralemmin downstream genes. The phylogenetic tree is shown in Figure 5. Although no sequence similar to AKAP2 was previously detected near *PALM1* [10], we could now find such genes downstream of all four main paralemmin isoform genes, with notable differential gene losses (see Results, Table 1). The chromosomal locations of the *PDG* genes as well as the phylogenetic analysis shows that the paralemmin downstream genes have evolved contiguously with the paralemmins. No *PDG* genes could be found in the lamprey nor the elephant shark genomes, which diminishes the phylogenetic resolution of this family.

In teleost fishes the chromosomal duplication in 3R that generated the A and B duplicates of *PALM1* and *PALMD* also gave rise to duplicates of the downstream genes to generate *PDG4A* and -*4B* flanking *PALMD-A* and –*B*. However, no corresponding *PDG* duplication could be observed for *PALM1-A* and -*B* since the *PDG* genes adjacent to *PALM1-B* appear to have been lost after 3R. The 3R event also generated duplicate *PDG3A* and -*B* genes. While the *PDG3A* genes are adjacent to the identified *PALM3* genes in all analyzed teleost genomes, the duplicate *PDG3B* sequences identified in stickleback and medaka are not adjacent to *PALM* genes (summarized in Figure 4). The analyses of the S1PR and PRG families (Figures S3 and S4 respectively) lend support for the generation of *PDG3A* and -*B* as part of a chromosomal block in 3R.

Consensus splice sites potentially allow alternative splicing to generate fusion proteins of *PALM1-PDG1* in tetrapods and *PALM1-A*-*PDG1A* in teleost fishes, but no such read-through ESTs could be identified in NCBIs dbEST database. However, three zebrafish ESTs of *PALMD-A*-*PDG4A* read-through transcripts were found (see Results), suggesting the expression of a fusion protein. In contrast, the *PDG4B* gene was found to be inverted relative to *PALMD-B* in all studied teleost genomes except zebrafish.

The region around the human *AKAP2 (PDG2)* amino acid sequence LVQNAIQ (a.a. 573–579) (see Figure S6), constitutes a *Protein kinase* A regulatory subunit-II (PKA-RII) binding site [39] and is highly conserved in the species orthologs of *PDG2* but not in *PDG1*, *PDG3* or *PDG4*. However, as the RII-binding sites of AKAPs have no clear consensus sequence other than the propensity to form an amphiphilic alpha-helix [40], PKA-RII binding of the other paralogs cannot be excluded by sequence analysis. The molecular and biological properties of AKAP2 and its paralogs, and the significance of the Palm2-AKAP2 fusion proteins, remain unknown. The sequence features described above may offer possibilities to explore the roles of these proteins.

### Conclusions

Our combined analysis of sequences and chromosome regions leads to the conclusion that the four tetrapod paralemmin genes arose by chromosome duplications in 2R before the origin of jawed vertebrates (Figure 6). Their retention suggests that each of these four paralemmins has a unique function. We have found a single putative paralemmin in the lancelet genome, but in no other invertebrate. Teleost fishes gained duplicates of *PALM1* and *PALMD* in 3R. The zebrafish has retained six of these paralemmin genes. Although no 3R duplicate of *PALM3* seems to have been conserved, several of the neighboring genes on the PALM3-bearing chromosome blocks have done so, including the paralemmin downstream genes (PDG).

As paralemmin-1, -2 and -3 are predominantly expressed in the brain and have the ability to induce filopodia and dendritic spines, they may be important for the development and plasticity of complex nervous systems. Differences in exon organization and important structural motifs suggest that the two fish palmdelphin duplicates have undergone functional changes. Thus, our analyses point to both ancient expansion of the paralemmin family, retention of the members over several hundred million years, and potential functional changes in some family members.

## Supporting Information

Table S1 **Identified** ***PALM*** **and neighboring gene family sequences.** The table includes the respective database identifiers used to predict the sequences, as well as chromosome locations and genome assembly information for all species investigated.
(XLS)

Figure S1 **Phylogenetic maximum likelihood tree of the polypyrimidine tract-binding protein (PTBP) family.** *PTBP* genes could be identified neighboring all *PALM* genes but *PALM3*. The phylogenetic analysis of this family, as well as the chromosomal data, are consistent with the phylogenetic analysis of the paralemmins (Figure 3) and our proposed duplication scheme (Figure 6). Our analyses also support the duplication of *PTBP2* and *PTBP1* genes in 3R, as part of the same chromosome blocks as *PALM1* and *PALMD*. Sequence designations are as follows: species abbreviation (see Methods), followed by chromosomal or genomic scaffold number and a symbol identifying the subtype, based on the phylogenetic analysis. Colors are applied as in Figure 3.
(TIF)

Figure S2 **Phylogenetic maximum likelihood tree of the calponin (CNN) family.** *CNN* genes could be identified neighboring all *PALM* genes but *PALM2*. The phylogenetic analysis of this family as well as the chromosomal data, are consistent with the phylogenetic analysis of the paralemmins (Figure 3) and our proposed duplication scheme (Figure 6). The presence of duplicate *CNN1* and *CNN3* genes in the zebrafish genome suggests duplication in 3R. However, the chromosomal data only supports such a duplication for the *CNN3* genes, here denominated *CNN3A* and *CNN3B* (see Figure 6 and Table S1). Since no duplicates could be identified in any other teleost fish genome, the phylogenetic data is inconclusive. Sequence designations are applied as in Figure S1. Colors are applied as in Figure 3.
(TIF)

Figure S3 **Phylogenetic maximum likelihood tree of the sphingosine-1-phosphatase related protein (S1PR) family.** Members of this family are also known as endothelial differentiation lysophosphatidic acid G-protein coupled receptors (EDG). Since no *S1PR*-like sequence could be identified in the investigated invertebrate genomes, this tree is presented as an un-rooted radial tree. The phylogenetic resolution is not as clear for this tree as for most other identified neighboring families, probably due to relatively low sequence identity within the family as well as independent gene duplications and translocations. Nonetheless, this tree suggests the divergence of four main branches early in vertebrate evolution and *S1PR* genes could be identified neighboring all paralemmin isoform genes in the tetrapod genomes, excepting the frog (*Xenopus tropicalis*) genome. This genome assembly is not mapped to chromosomes and the *S1PR* genes are positioned in different chromosomal scaffolds than the paralemmin genes. The phylogenetic analysis as well as the chromosomal data also suggest that *S1PR2* and *S1PR5* arose as local duplicates on the *PALM3*-bearing chromosome block after 2R, and that *S1PR5* conserves duplicates from the 3R event, here called *S1PR5-A* and *S1PR5-B*. The chromosome locations of the identified *S1PR* genes in the teleost genomes suggest several gene translocation events in this lineage. Sequence designations are applied as in Figure S1. Colors are applied as in Figure 3.
(TIF)

Figure S4 **Phylogenetic maximum likelihood tree of the plasticity related gene (PRG) family.** Members of this family are also known as lipid phosphate phosphatase-related proteins (LPPR). *PRG* genes can be found neighboring all *PALM* isotype genes in the analyzed genomes. However, the topology of the resulting tree is not fully consistent with the paralemmin trees (Figure 3), likely due to a local duplication event before the 2R events. This is consistent with the chromosomal data and our proposed duplication scheme (Figure 6). The tree is rooted with an identified *C. elegans* family member to provide a better relative dating for this event. The phylogenetic analysis and chromosomal data taken together also support the duplication of *PRG1* and *PRG5* genes in 3R as part of the same chromosome block as *PALMD*, as well as of PRG2 and PRG4 as part of the same chromosome blocks as *PALM1* and *PALM3* respectively. One putative *PRG* sequence was identified in the lamprey genome: Although the phylogenetic analysis is inconclusive as to its identity due to the low statistical support within the branch, it seems to be more similar to the *PRG1* and *PRG2* family members. Two putative *PRG* sequences were identified in the lancelet genome, however their identity is not resolved in the phylogenetic analysis. It's possible that they represent an independent duplication in the lancelet lineage. The identified *PRG2*-like sequence in chicken and *PRG4A*-like sequence in stickleback were not included in the phylogenetic analysis due to poor sequence quality in the genome databases. Sequence designations are applied as in Figure S1. Colors are applied as in Figure 3.
(TIF)

Figure S5 **Phylogenetic maximum likelihood tree of the ATP-binding cassette sub-family A (ABCA) family.** *ABCA* genes could be identified neighboring all *PALM* genes except *PALM3*. Taken together the phylogenetic analysis and the chromosomal data are consistent with the phylogenetic analysis of the paralemmins (Figure 3) and our proposed duplication scheme (Figure 6). Our analyses also support the duplication of *ABCA4* genes in 3R, as part of the same chromosome block as *PALMD*. The identified *ABCA7*-like sequence in chicken was not included in the phylogenetic analysis due to poor sequence quality in the genome database. Sequence designations are applied as in Figure S1. Colors are applied as in Figure 3.
(TIF)

Figure S6 **Sequence alignment of the paralemmin-downstream gene (PDG) family.** These sequences could be identified next to *PALM2* (*PDG2/AKAP2*), *PALM1* (*PDG1*), *PALM3* (*PDG3A* and -*B*), *PALMD* (*PDG4*), *PALMD-A* (*PDG4A*) and *PALMD-B* (*PDG4B*). The RII binding site of AKAP2 at positions 878–896, and the C-terminal PDG motif conserved in all isoforms, are marked by boxes above the alignment.
(TIF)
