# Supplementary figures and images for: Evolution of the Vertebrate Paralemmin Gene Family: Ancient Origin of Gene Duplicates Suggests Distinct Functions
Source: PLoS One. 2012 Jul 25;7(7):e41850. doi: 10.1371/journal.pone.0041850 (PMC3405040; doi:10.1371/journal.pone.0041850)

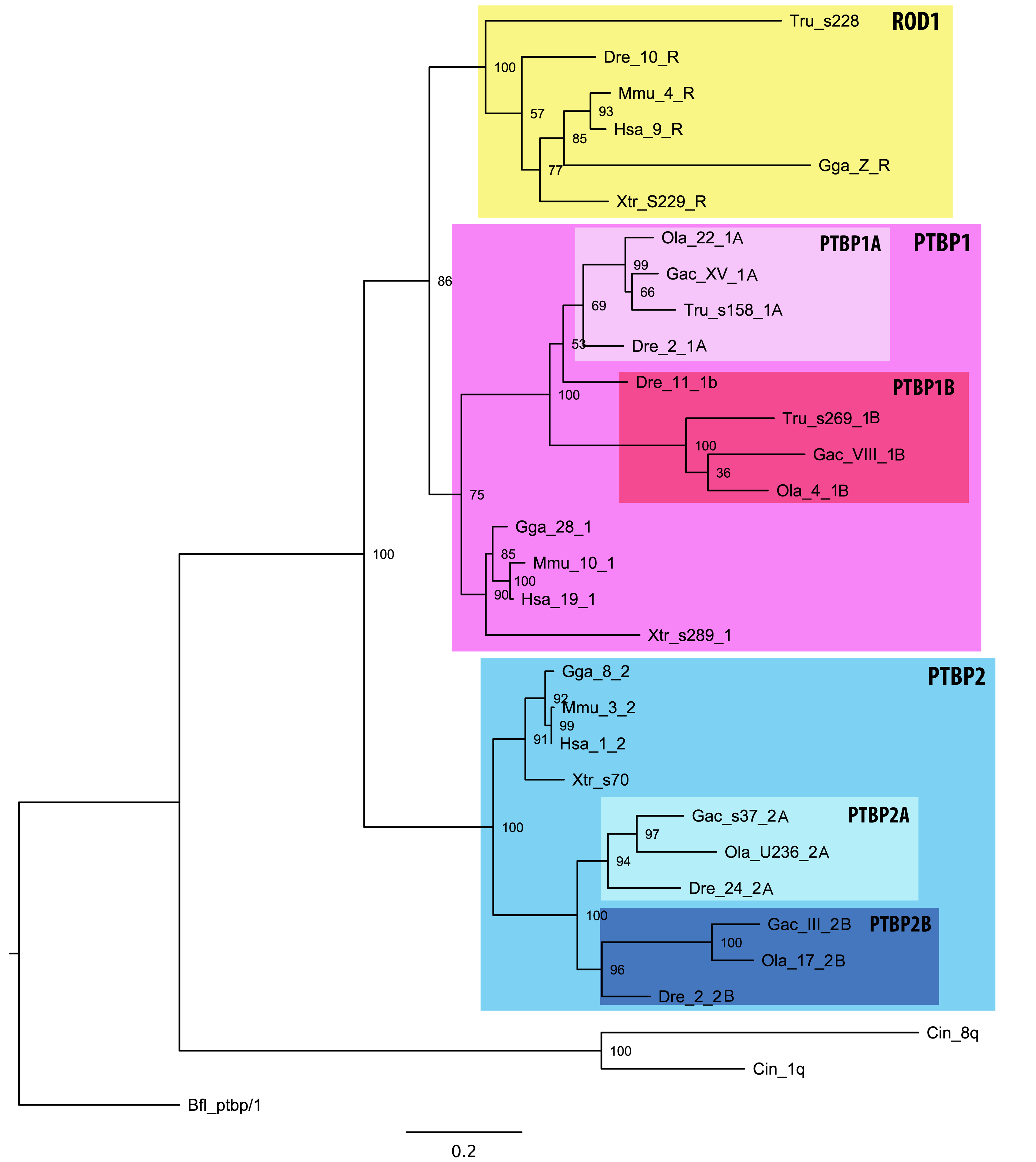

Supplement: Figure S1 — Phylogenetic maximum likelihood tree of the polypyrimidine tract-binding protein (PTBP) family. PTBP genes could be identified neighboring all PALM genes but PALM3. The phylogenetic analysis of this family, as well as the chromosomal data, are consistent with the phylogenetic analysis of the paralemmins (Figure 3) and our proposed duplication scheme (Figure 6). Our analyses also support the duplication of PTBP2 and PTBP1 genes in 3R, as part of the same chromosome blocks as PALM1 and PALMD. Sequence designations are as follows: species abbreviation (see Methods), followed by chromosomal or genomic scaffold number and a symbol identifying the subtype, based on the phylogenetic analysis. Colors are applied as in Figure 3. (TIF) [file pone.0041850.s002.tif]

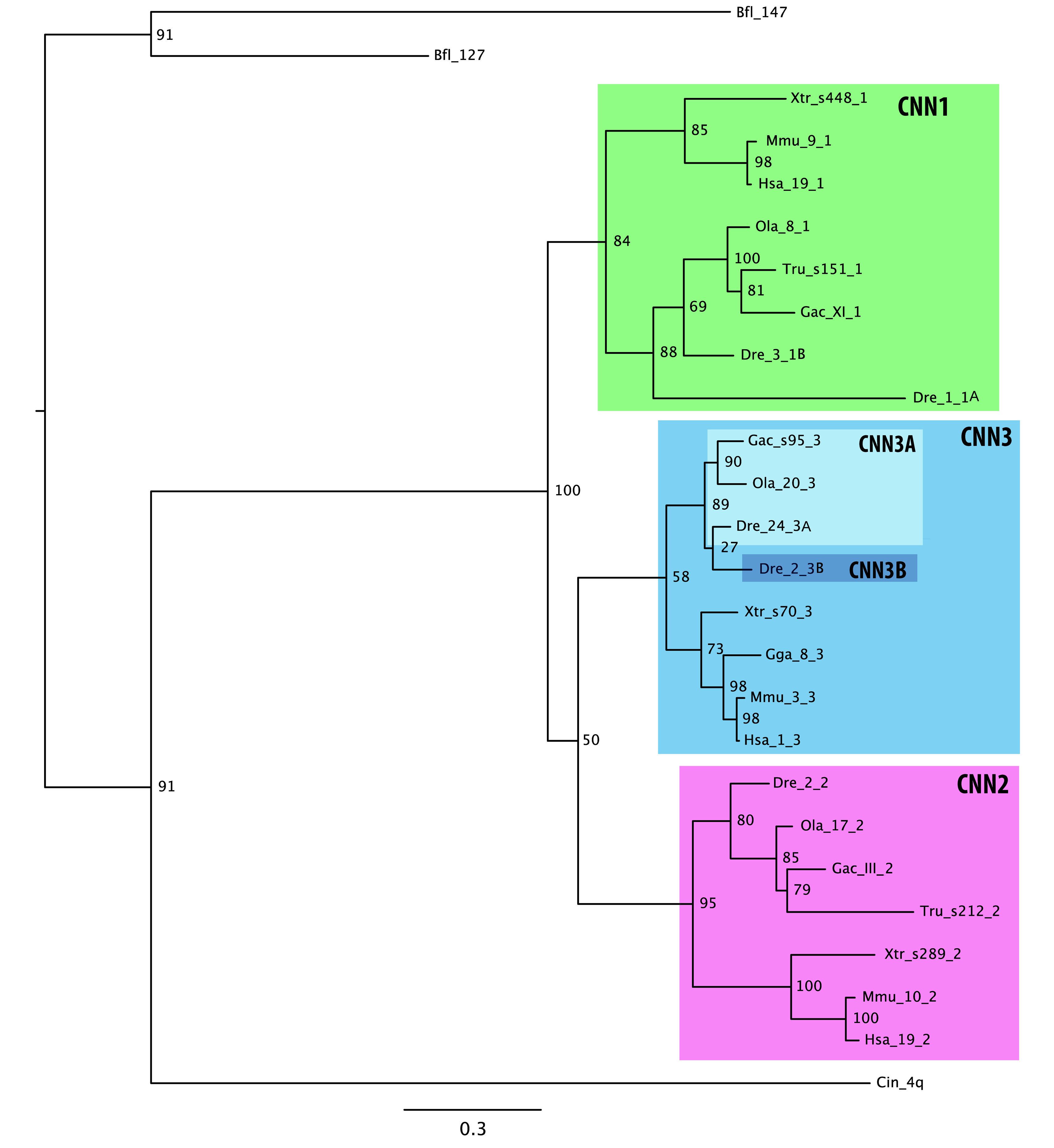

Supplement: Figure S2 — Phylogenetic maximum likelihood tree of the calponin (CNN) family. CNN genes could be identified neighboring all PALM genes but PALM2. The phylogenetic analysis of this family as well as the chromosomal data, are consistent with the phylogenetic analysis of the paralemmins (Figure 3) and our proposed duplication scheme (Figure 6). The presence of duplicate CNN1 and CNN3 genes in the zebrafish genome suggests duplication in 3R. However, the chromosomal data only supports such a duplication for the CNN3 genes, here denominated CNN3A and CNN3B (see Figure 6 and Table S1). Since no duplicates could be identified in any other teleost fish genome, the phylogenetic data is inconclusive. Sequence designations are applied as in Figure S1. Colors are applied as in Figure 3. (TIF) [file pone.0041850.s003.tif]

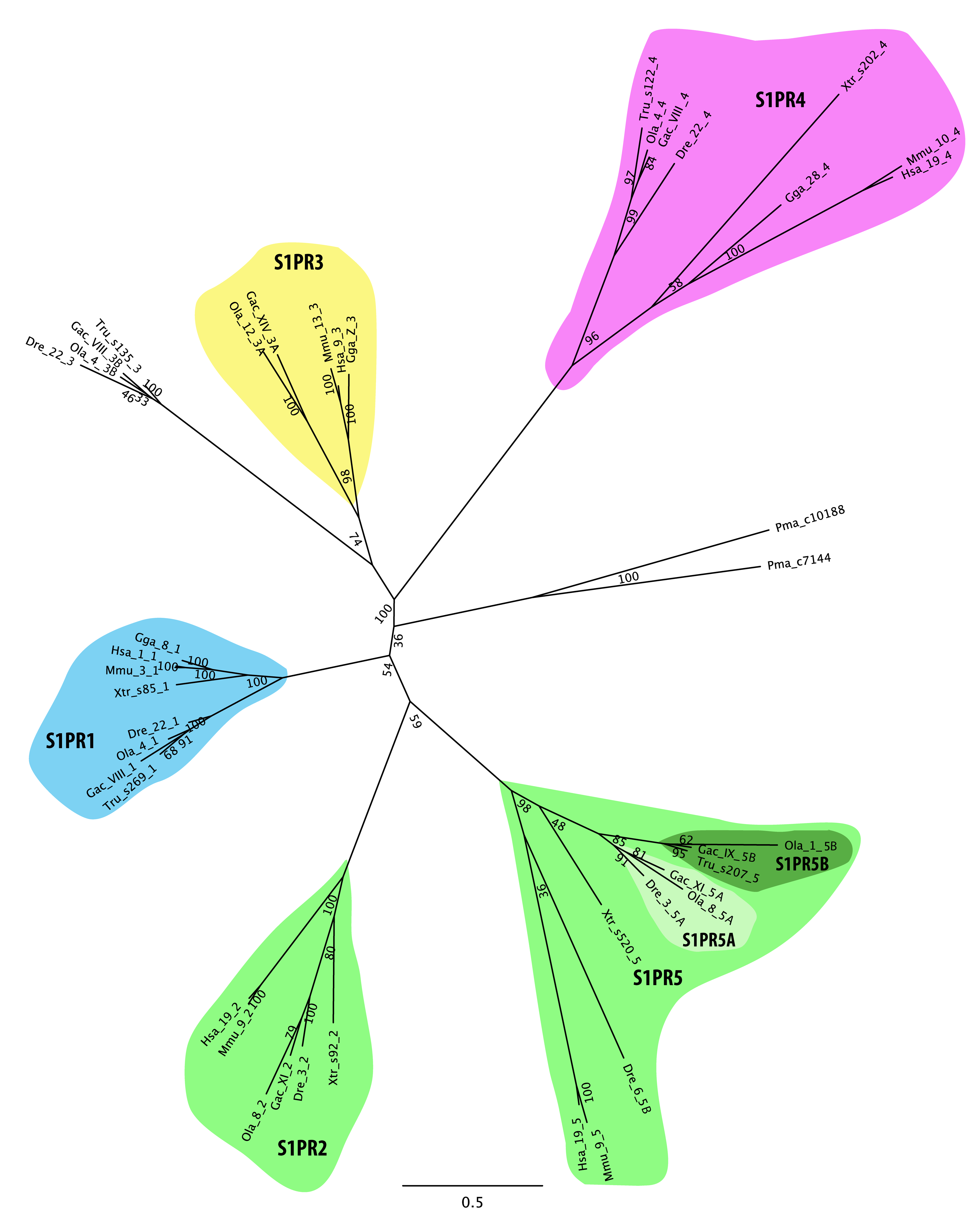

Supplement: Figure S3 — Phylogenetic maximum likelihood tree of the sphingosine-1-phosphatase related protein (S1PR) family. Members of this family are also known as endothelial differentiation lysophosphatidic acid G-protein coupled receptors (EDG). Since no S1PR-like sequence could be identified in the investigated invertebrate genomes, this tree is presented as an un-rooted radial tree. The phylogenetic resolution is not as clear for this tree as for most other identified neighboring families, probably due to relatively low sequence identity within the family as well as independent gene duplications and translocations. Nonetheless, this tree suggests the divergence of four main branches early in vertebrate evolution and S1PR genes could be identified neighboring all paralemmin isoform genes in the tetrapod genomes, excepting the frog (Xenopus tropicalis) genome. This genome assembly is not mapped to chromosomes and the S1PR genes are positioned in different chromosomal scaffolds than the paralemmin genes. The phylogenetic analysis as well as the chromosomal data also suggest that S1PR2 and S1PR5 arose as local duplicates on the PALM3-bearing chromosome block after 2R, and that S1PR5 conserves duplicates from the 3R event, here called S1PR5-A and S1PR5-B. The chromosome locations of the identified S1PR genes in the teleost genomes suggest several gene translocation events in this lineage. Sequence designations are applied as in Figure S1. Colors are applied as in Figure 3. (TIF) [file pone.0041850.s004.tif]

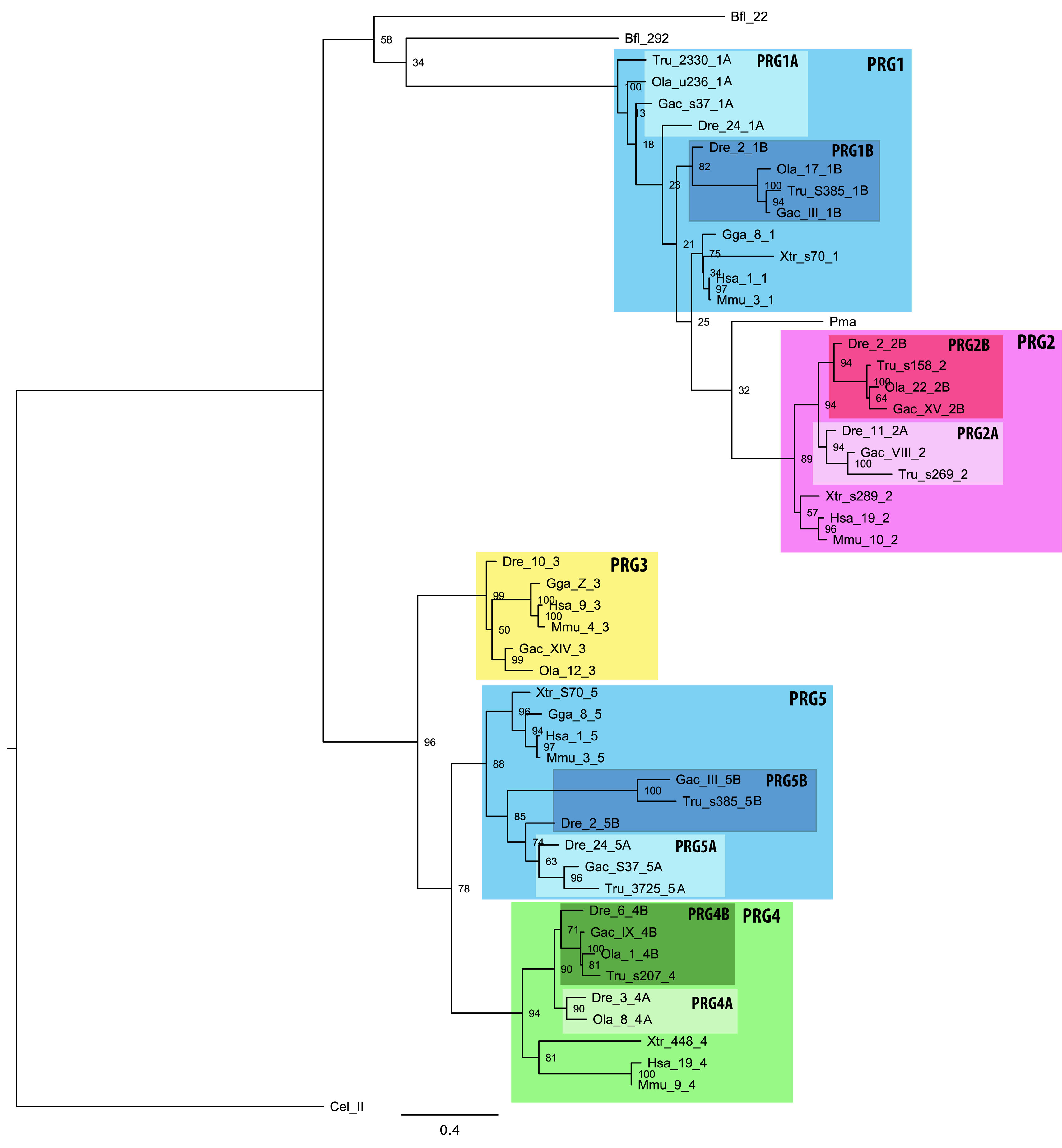

Supplement: Figure S4 — Phylogenetic maximum likelihood tree of the plasticity related gene (PRG) family. Members of this family are also known as lipid phosphate phosphatase-related proteins (LPPR). PRG genes can be found neighboring all PALM isotype genes in the analyzed genomes. However, the topology of the resulting tree is not fully consistent with the paralemmin trees (Figure 3), likely due to a local duplication event before the 2R events. This is consistent with the chromosomal data and our proposed duplication scheme (Figure 6). The tree is rooted with an identified C. elegans family member to provide a better relative dating for this event. The phylogenetic analysis and chromosomal data taken together also support the duplication of PRG1 and PRG5 genes in 3R as part of the same chromosome block as PALMD, as well as of PRG2 and PRG4 as part of the same chromosome blocks as PALM1 and PALM3 respectively. One putative PRG sequence was identified in the lamprey genome: Although the phylogenetic analysis is inconclusive as to its identity due to the low statistical support within the branch, it seems to be more similar to the PRG1 and PRG2 family members. Two putative PRG sequences were identified in the lancelet genome, however their identity is not resolved in the phylogenetic analysis. It's possible that they represent an independent duplication in the lancelet lineage. The identified PRG2-like sequence in chicken and PRG4A-like sequence in stickleback were not included in the phylogenetic analysis due to poor sequence quality in the genome databases. Sequence designations are applied as in Figure S1. Colors are applied as in Figure 3. (TIF) [file pone.0041850.s005.tif]

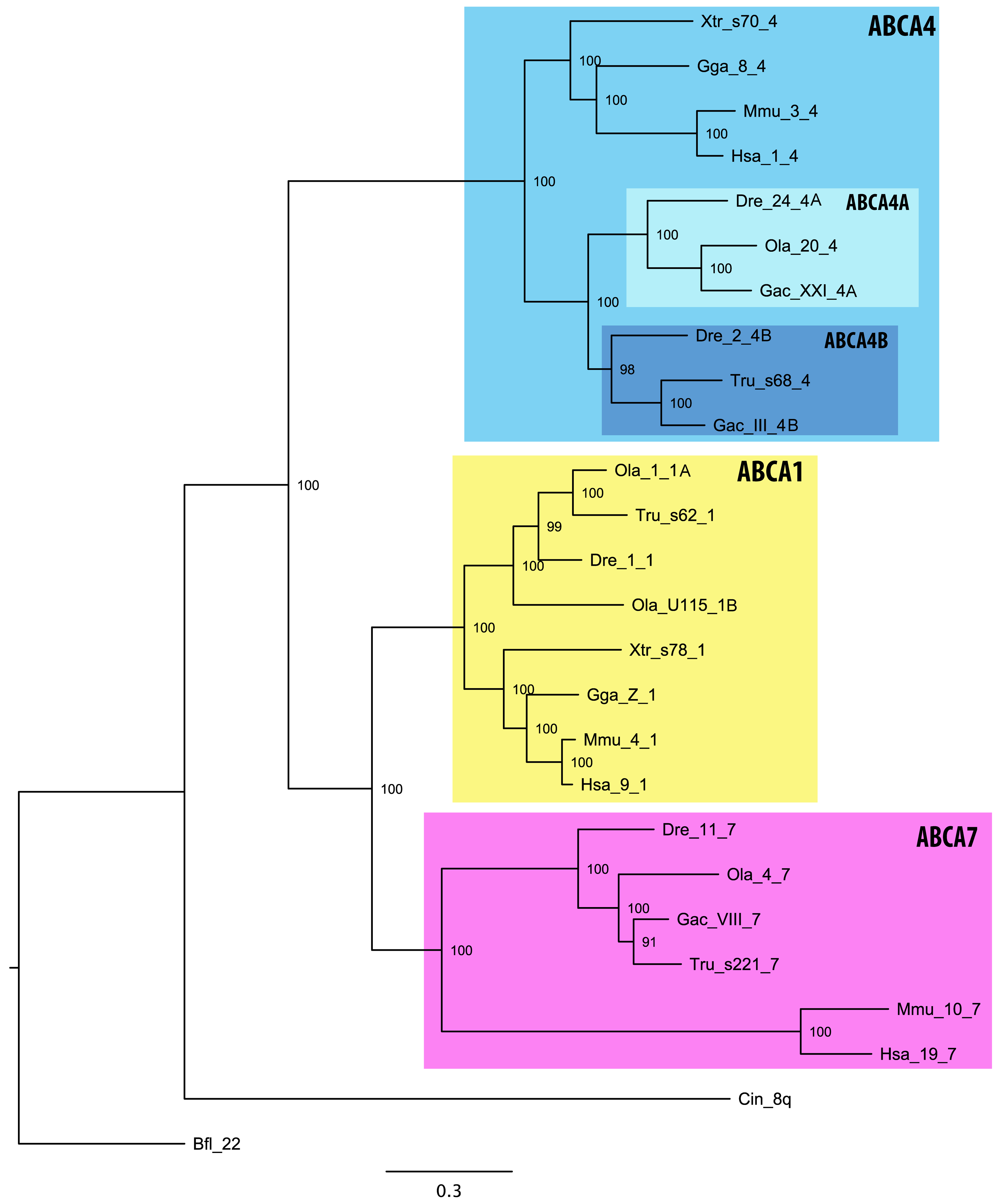

Supplement: Figure S5 — Phylogenetic maximum likelihood tree of the ATP-binding cassette sub-family A (ABCA) family. ABCA genes could be identified neighboring all PALM genes except PALM3. Taken together the phylogenetic analysis and the chromosomal data are consistent with the phylogenetic analysis of the paralemmins (Figure 3) and our proposed duplication scheme (Figure 6). Our analyses also support the duplication of ABCA4 genes in 3R, as part of the same chromosome block as PALMD. The identified ABCA7-like sequence in chicken was not included in the phylogenetic analysis due to poor sequence quality in the genome database. Sequence designations are applied as in Figure S1. Colors are applied as in Figure 3. (TIF) [file pone.0041850.s006.tif]

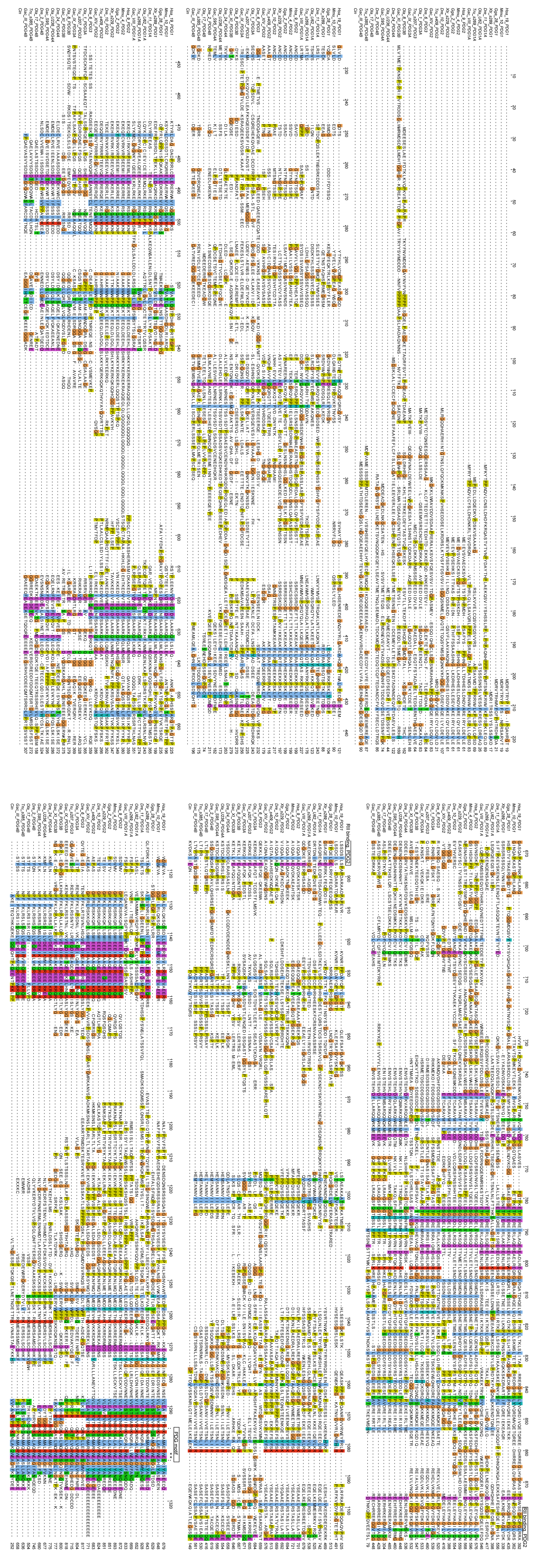

Supplement: Figure S6 — Sequence alignment of the paralemmin-downstream gene (PDG) family. These sequences could be identified next to PALM2 (PDG2/AKAP2), PALM1 (PDG1), PALM3 (PDG3A and -B), PALMD (PDG4), PALMD-A (PDG4A) and PALMD-B (PDG4B). The RII binding site of AKAP2 at positions 878–896, and the C-terminal PDG motif conserved in all isoforms, are marked by boxes above the alignment. (TIF) [file pone.0041850.s007.tif]
